# P30 - Could a hydrolysed rice protein formula induce sensitisation to rice protein?

**DOI:** 10.1186/2045-7022-4-S1-P85

**Published:** 2014-02-28

**Authors:** Delphine De Boissieu, Juliette Rouzière

**Affiliations:** 1 Hopital Necker, Paris, France

## 

Previous studies have shown that hydrolysed rice protein formula (HRPF) is well tolerated in infants with cow’s milk allergy (CMA). Children receiving HRPF showed similar growth and development of clinical tolerance to those receiving an extensively hydrolysed cow’s milk protein (eHF) formula.

We present 3 cases of infants in which CMA symptoms persisted with HRPF feeding, and resolved with an amino-acid based formula (AAF). In all 3 cases, patients had a non IgE mediated CMA with prick test and specific IgE negative for milk and a positive milk atopy patch test.

Case 1 was a breast-fed boy with a typical food protein induced enterocolitis syndrome (FPIES) with cow’s milk formula. He was fed with an AAF without symptoms. At age 12 months, a HRPF was introduced and FPIES developed with this formula.

Case 2 was a boy with atopic dermatitis during exclusive breast-feeding. At age 4 months, feedings of an HRPF were introduced episodically. At 41/2 months, the child presented to clinic with a typical FPIES (an acute episode of vomiting, lethargy and pallor) after drinking a bottle of HRPF. Breast-feeding was prolonged and an AAF introduced with success.

Case 3 was a 3-month old boy who presented with gastro-oesophageal reflux associated with a severe food impaction during exclusive breast-feeding. These symptoms disappeared with CMP exclusion in the mother’s diet. At age 4 months, a HRPF was introduced and 15 days later, the infant presented with gastro-oesophageal reflux, difficulty feeding and failure to thrive. The formula was changed to an AAF and symptoms disappeared in a few days.

The results of the atopy patch test are presented in the table below. A sensitisation to rice was observed in case 1 and 2, but parents denied a challenge with rice.

**Table 1:** Results of the atopy patch test

Atopy patch test	With cow’s milk	With HRPF	With native rice
Case 1	Positive	Positive	Positive

Case 2	Positive	Not done	Positive

Case 3	Positive	Positive	Negative

## Conclusions

Some children with non-IgE mediated CMA and digestive symptoms do not tolerate HRPF and must be fed with an AAF. More studies are necessary to determine whether the HRPF induces only a sensitization or a real rice allergy.

